# Measurement-device-independent quantum key distribution via quantum blockade

**DOI:** 10.1038/s41598-018-21576-7

**Published:** 2018-03-07

**Authors:** Yi-Heng Zhou, Zong-Wen Yu, Ao Li, Xiao-Long Hu, Cong Jiang, Xiang-Bin Wang

**Affiliations:** 1 Department of Physics, State Key Laboratory of Low Dimensional Quantum Physics, Tsinghua University, Beijing, 100084 People’s Republic of China; 2 Synergetic Innovation Center of Quantum Information and Quantum Physics, University of Science and Technology of China, Hefei, Anhui 230026 China; 3 Data Communication Science and Technology Research Institute, Beijing, 100191 China; 4 Jinan Institute of Quantum technology, SAICT, Also a member of Center for Atomic and Molecular Nanosciences at Tsinghua University, Jinan, 250101 People’s Republic of China

## Abstract

Efficiency in measurement-device-independent quantum key distribution(MDI-QKD) can be improved not only by the protocol, but also single-photon sources. We study the behavior of MDI-QKD with statistical fluctuation using quantum blockade source. Numerical simulation for a type of 4-intensity protocol shows that, after parameter optimization, this source can improve the final key rate by 100 times compared with traditional weak coherent state sources.

## Introduction

Quantum key distribution (QKD)^1–5^ allows two remote parties to distribute secure keys through public channels. QKD can ensure unconditional security guaranteed by the laws of quantum physics^1–5^. However, in practical implementation, the imperfections of QKD system like imperfect single-photon sources will cause loopholes that eavesdropper may make use of. To overcome this insecurity, the decoy state method was proposed^6–26^, and one can still keep the unconditional security of QKD with imperfect single-photon sources^27–31^. Then, to patch up the loophole caused by the limitation of detection efficiency as well as channel losses^32,33^, the device independent QKD (DI-QKD)^34–37^ and the measurement-device independent QKD (MDI-QKD)^38–40^ were developed. The combination of the decoy-state method and MDI-QKD has been studied both experimentally^41–45^ and theoretically^46–57^.

Yet, because of the complexity of the system compared with the BB84 protocol, the key rate of the decoy-state MDI-QKD is rather lower than BB84. Taking into account the statistical fluctuation, the data size and the communication time become the great influence to the final key rate^47,48,50,56^. For example, the number of total pulses at each side *N* is usually larger than 10^12^ to generate keys. To overcome this difficulty, previously^58^ we provided a very efficient 4-intensity protocol which can remarkably improve the key rate and communication distance. It can improve the key rate by almost 2 magnitude orders after parameter optimization. However, one should not counted on the unrealistic wish to further improve the efficiency unlimitedly by taking progress on the protocol only. We should also consider the path of improving the quality of the single-photon sources. Generally, at present the weak coherent state(WCS) sources are used for practical QKD. But the WCS pulses have a large vacuum component and significant fraction of multi-photon states, these will severely reduce the key rate and transmission distance. The situation is even worse in MDI-QKD because the large fraction of vacuum pulses(both vacuum decoy states and vacuum fraction in a Poisson pulse) at one side lead to a large observed value of the error rate in *X* basis. Fortunately, a quantum blockade source(QBS) can conspicuously enhance the signal photon component. The method of using quantum blockade source in QKD was proposed^59^ with a simple simulation of asymptotic key rate. However, it considers only the traditional BB84 protocol, it has not studied the important MDI-QKD and the finite size effects for practical QKD. Here in this work, we study the application of quantum blockade source in practical decoy state method MDI-QKD with most high efficient protocol, the 4-intensity protocol. Our numerical results will verify the remarkable progress of about 100 times rise in key rate.

## Results

### The photon blocade

Photon blockade as a nonlinear quantum optical process can be realized experimentally with single atoms coupled to a resonator^60,61^, solid-state with (quantum dot) QD coupled to dielectric resonators^62–65^ and nonlinear Kerr medium^66,67^. Particularly, Kerr-type material has advantages in its controllability and flexibility. To obtain a high quality single-photon sources, in our previous work(OL2016), we have calculated and simulated explicitly the photon-number distribution for pulses outside cavities. In the single-photon blockade using Kerr-type resonators under the condition of different parameters, it reveals an optimized single-photon state probability. In that work, we have simulated the system with quantum trajectory method^68–70^. In this method, for each single trajectory we simulate, we monitory the number of photons from the output of the resonantor. And the output light could be generally expanded in the Fock basis as: $|{a}_{out}\rangle ={\sum }_{n=0}^{\infty }{c}_{n}|n\rangle$ where |*c*_*n*_|^2^ is the probability of photon state |*n*〉. At last we can estimate the photon number probability *P*_*n*_ = $\frac{{|{c}_{n}|}^{2}}{\sum {|{c}_{i}|}^{2}}$ from the number of counted photons.

Obviously, when evaluating the superiority of certain sources in decoy state method QKD, the photon number probability is wanted. To obtain *P*_*n*_, one needs to simulate the system using quantum trajectory method. For this, the calculation takes a lot of computation resource. And it is not likely to reach the continuous functions but discrete results (seen in Table 1) of the system parameters to *P*_*n*_. This situation urges us to change the normal strategy of dealing with decoy state method MDI-QKD under the influence of statistical fluctuation, which will be discussed later in the work.

**Table 1:** The photon number probability of two typical different sets of system parameters in ref.^59^.

	*P*_0_	*P*_1_	*P*_2_	*P*_3_
I	39.15%	47.38%	12.88%	0.06%
II	25.75%	67.92%	6.30%	0.03%
III	24.17%	71.37%	4.42%	0.05%

### Protocol

We use subscript *A* or *B* to denote a source at Alice’s side or Bob’s side. In the protocol we proposed before^58^, sources *x*_*A*_ and *y*_*A*_ (*x*_*B*_ and *y*_*B*_) only emit pulses in *X* basis while source *z*_*A*_ (*z*_*B*_) only emits pulses in *Z* basis. The protocol needs four different states ${\rho }_{{o}_{A}}=|0\rangle \langle 0|,{\rho }_{{x}_{A}},{\rho }_{{y}_{A}},{\rho }_{{z}_{A}}$ $({\rho }_{{o}_{B}}=|0\rangle \langle 0|,{\rho }_{{x}_{B}},{\rho }_{{y}_{B}},{\rho }_{{z}_{B}})$ respectively.

In photon number space, suppose

1 $${\rho }_{{x}_{A}}=\sum _{k}{a}_{k}|k\rangle \langle k|,\quad {\rho }_{{x}_{B}}=\sum _{k}{b}_{k}|k\rangle \langle k|,$$

2 $${\rho }_{{y}_{A}}=\sum _{k}a{^{\prime} }_{k}|k\rangle \langle k|,\quad {\rho }_{{y}_{B}}=\sum _{k}b{^{\prime} }_{k}|k\rangle \langle k|,$$

3 $${\rho }_{{z}_{A}}=\sum _{k}a{^{\prime\prime} }_{k}|k\rangle \langle k|,\quad {\rho }_{{z}_{B}}=\sum _{k}b{^{\prime\prime} }_{k}|k\rangle \langle k|,$$

We call *x*_*A*_, *x*_*B*_ as well as *y*_*A*_, *y*_*B*_ the decoy sources; *z*_*A*_, *z*_*B*_ the signal sources, and *o*_*A*_, *o*_*B*_ the vacuum sources.

At each time, Alice will randomly choose source *l*_*A*_ with probability ${p}_{{l}_{A}}$ for *l* = *o*, *x*, *y*, *z*. Similarly, Bob will randomly choose source *r*_*B*_ with probability ${p}_{{r}_{B}}$ for *r* = *o*, *x*, *y*, *z*. The emitted pulse pairs (one pulse from Alice, one pulse from Bob) are sent to the un-trusted third party (UTP). We shall use notation *lr* to indicate the two-pulse source when Alice use source *l*_*A*_ and Bob use source *r*_*B*_ to general a pulse pair, e.g., source *xy* is the source that Alice uses source *x*_*A*_ and Bob uses source *y*_*B*_. Also, here in our protocol, the intensity for pulses in *Z* basis can be different from those of *X* basis, this makes more freedom in choosing the intensities and hence further raises the key rate. Those effective events caused by pulse pairs from source *zz* will be used for key distillation, while the effective events caused by sources in *X* basis and vacuum sources will be used to estimate the yield and the phase-flip error rate of the single-photon pulse pairs.

The final key rate of per pulse pair can be calculated as^6,7^

4 $$\begin{array}{l}R={p}_{{z}_{A}}{p}_{{z}_{B}}\cdot \{{a}_{1}^{\text{'}\text{'}Z}{b}_{1}^{\text{'}\text{'}Z}{\underline{s}}_{11}\mathrm{[1}-H({\bar{e}}_{11})]-f{S}_{zz}H({E}_{zz})\},\end{array}$$

In which, ${\underline{s}}_{11}$ is the lower bound of the single photon counting rate *s*_11_, and ${\bar{e}}_{11}$ is the upper bound of the single photon error rate *e*_11_, *H* is the binary Shannon entropy, *f* is the factor of error correction inefficiency. *S*_*lr*_ note the counting rate in UTP while Alice choice the source *l* and Bob choice the source *r*, *E*_*lr*_ and *T*_*lr*_ note the error rate and error counting rate respectively. (Meaning *T*_*lr*_ = *E*_*lr*_*S*_*lr*_).

As *S*_*zz*_ and *E*_*zz*_ can directly get in UTP, to obtain the final key rate, one needs to know ${\underline{s}}_{11}$ as well as ${\bar{e}}_{11}$ by the decoy state method. As was shown in ref.^58^, both ${\underline{s}}_{11}$ and ${\bar{e}}_{11}$ are functionals of a common variable ${\mathcal H}$ (See details in the appendix). The final key rate is then

5 $$R=\mathop{{\rm{\min }}}\limits_{ {\mathcal H} \,\in \, {\mathcal I} } {\mathcal R} ( {\mathcal H} \mathrm{).}$$

And as shown in detail in the appendix, ${\mathcal I}$ is the range of values for ${\mathcal H}$.

### Numerical simulation

With the protocol introduced above, we can numerically calculate the key rate and evaluate the performance of the MDI-QKD. In considering the finite-size effects, we shall take a failure probability of 10^−7^ with a normal distribution. Finite size effects are very important in the practical application of QKD, because of the finite transition time and relatively small data size, especially when one needs communication with little delay, like generating fresh key.

For these purpose, to achieve a practical useful key rate, we need both the protocol introduced above and the optimization algorithm. There are variety parameters in the protocol (if using the traditional WCS sources, the parameters are the intensities and the emitting probabilities for each sources, which means six variables). Not like the situation without statistical fluctuation, globally optimization will make remarkable difference in the final key rates.

Usually, we describe the decoy state method system by several continuously parameters and optimiz them in computer program. But the case here makes this strategy difficult, because our quantum blockade source is described by the disperse *P*_*n*_. Though these *P*_*n*_ are essentially based on several continuously parameters in quantum blockade system, but as discuss above, one have to implement Monte Carlo algorithm to conduct the photon number distribution, which takes a lot of time and the results are may not smooth enough for the farther optimization.

To solve the predicament, we no longer optimize the quantum blockade source itself, but add a linear attenuation device(optical fiber for example) right after each source. Equally obtain the continuously changing source as

6 $$P{^{\prime} }_{n}=\sum _{k=n}^{\infty }{P}_{k}{\eta }^{n}{\mathrm{(1}-\eta )}^{k-n}{C}_{k}^{n}$$

here *η* is the penetration rate, and *η* ∈ [0, 1].

Through this treatment, we need only one kind of quantum blockade source to accomplish the whole decoy state method MDI-QKD. By choice three different *η*, two decoy sources and one signal source can be easily obtained. And we just need to optimize the three *η* and corresponding emittion probabilities, which would make the problem simpler and more calculable.

In the numerical simulation, we take a simple treatment using normal distribution to make a fair comparison with the prior art results^56–58^, and uniformly set failure probability *ε* = 10^−7^, and implement the global optimization for each method compared in our figures.

Table 2 shows the device parameters and data sizes used in numerical simulations. Except for the parameters listed, we also set error correction inefficiency *f*_*e*_ = 1.16 for all the simulation. The parameters choice is based on^58^ to provide comprehensive and fair comparison and the two lines represent two typical experimental setup.

**Table 2:** Device parameters and data sizes used in numerical simulations. *e*_*d*_: the alignment error. *p*_*d*_: the dark count rate. *η*_*d*_: the detection efficiency of all detectors. *N*: the total pulse pair emitted.

	*e*_*d*_	*p*_*d*_	*ηd*	*N*
*a*	1.5%	6.02 × 10^−6^	14.5%	10^10^
*b*	1%	10^−7^	40%	10^13^

**Figure 1:**
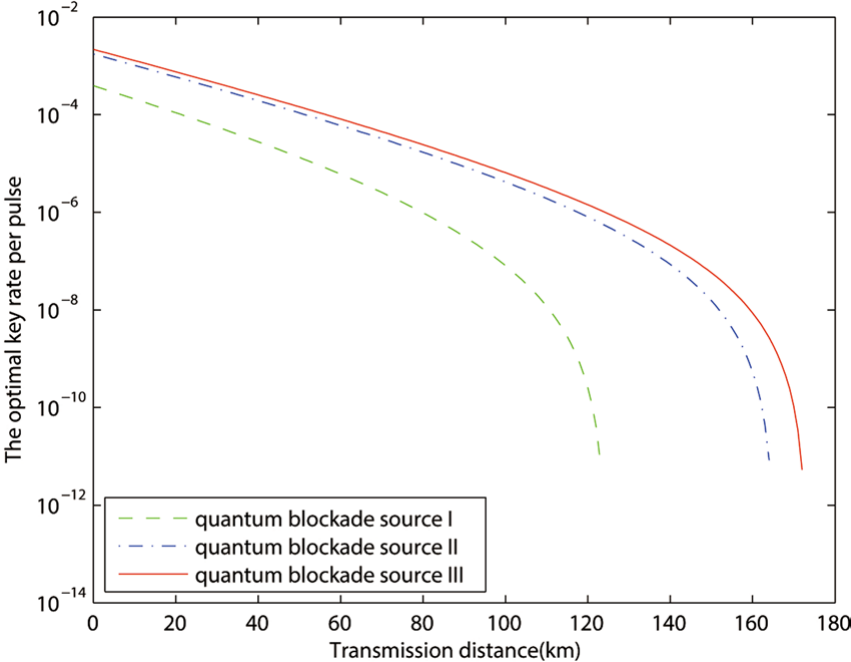
The optimized key rates (per pulse pair) versus transmission distance by different sources with device parameters and data size being given by line *a* of Table 2. The quantum blockade source is choice from Table 1.

In Fig. 1, we compare the performances of quantum blockade source with different photon number distributions in Table 1. The result approximately declares that *P*_1_ is the most important parameter to estimate the advantage of quantum blockade source in MDI-QKD. But we must point out that the key rate is also affect by the other *P*_*n*_.

**Figure 2:**
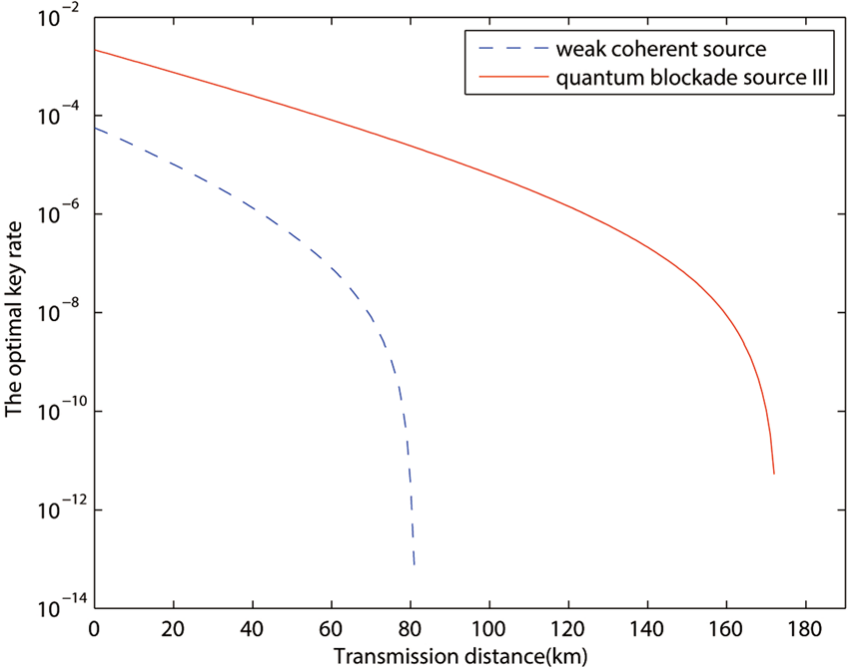
The optimized key rates (per pulse pair) versus transmission distance by different sources with device parameters and data size being given by line *a* of Table 2.

**Figure 3:**
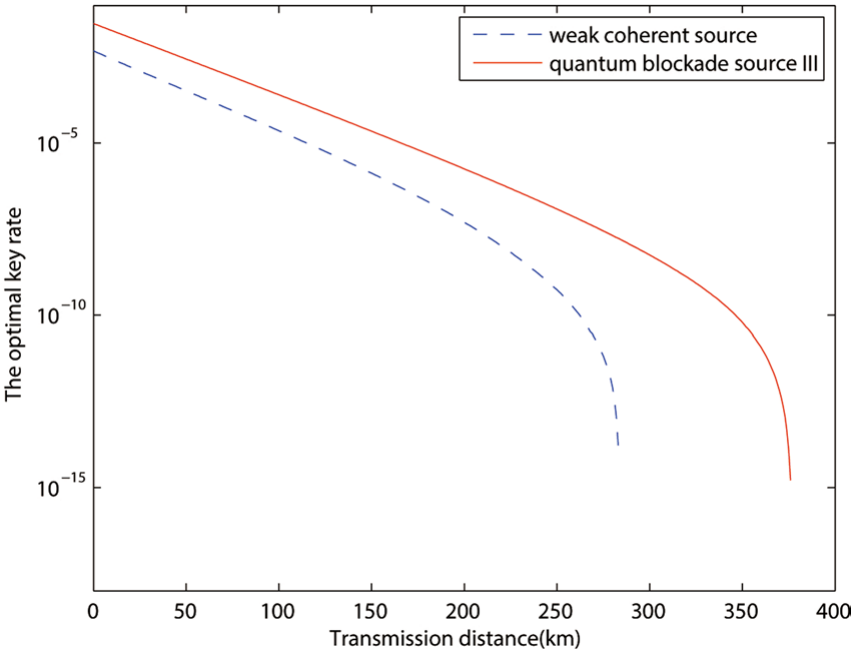
The optimized key rates (per pulse pair) versus transmission distance by different sources with device parameters and data size being given by line *b* of Table 2.

**Figure 4:**
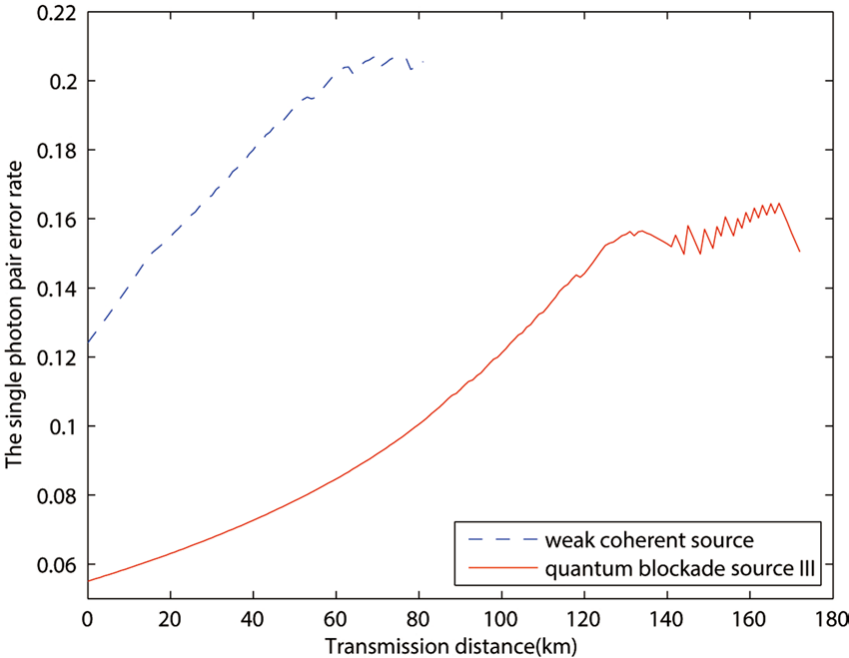
The single photon pair error rates versus transmission distance by different sources of the calculation in Fig. 1.

Based on this conclusion, in Figs 2 and 3, we chooses quantum blockade source III to compare with the traditional weak coherent source, and the advantage is observable with both case a and case b. In Fig. 4, we plot the single photon pulse pair error rates of the two sources discussed above in the calculation of case a in Fig. 2. And it clearly shows that the remarkable reduction of single photon pulse pair error rate for the quantum blockade source comparing with the traditional weak coherent source, which is the main contribution to the improvement of key rate.

We also give some typical key rates in certain distance points in Table 3. In that table, the key rates in lines 2 and 3 are obtained with parameters of case a, and the other two lines are calculated with parameters of case b in Table 2. It clearly shows the significantly increasing of the key rate due to the quantum blockade sources used, and we also explain that this advantage is mainly due to the reduction of single photon pair error rate.

**Table 3:** The key rates of some typical distance points. The line two and line three are the simulation of Fig. 2, the line four and line five are the simulation of Fig. 3.

	50 *km*	70 *km*
weak coherent source	3.78 × 10^−7^	8.10 × 10^−9^
quantum blockade source	1.45 × 10^−4^	4.49 × 10^−5^
weak coherent source	3.31 × 10^−4^	1.15 × 10^−4^
quantum blockade source	2.77 × 10^−3^	1.07 × 10^−3^

And here we also compare the secure key rate of our numerical simulation with an existing MDI-QKD experiment^71^. Given the same detector parameters, alignment errors and pulse number, at the distance of 259 *km*, the key rate of the MDI-QKD experiment with weak coherent state is 3.48 × 10^−9^ and the key rate of quantum blockade sources is 1.88 × 10^−7^.

## Discussion

In summary, we have investigated the performance of quantum blockade source in practical MDI-QKD. Although the previous work^59^ obtain the similar simulations to prove the advantage of quantum blockade source comparing with the WCS, but the statistical fluctuation and global optimization are not included there. Even the finite data size reduces the key rate, one can still reach the irresistibly superiority of quantum blockade source by the efficient decoy state method considering statistical fluctuation^58^ and global optimization strategy. It demonstrates that by implementing the scheme above, the quantum blockade source can greatly improve the key rate and communication distance of practical MDI-QKD, which is nearly tens of or hundreds of times.

## Methods

When statistical fluctuation is considered, the estimation will be tougher, but as mentioned above, using our newly developed strategy^58^, those two parameters of great importance can be estimated easily and tight.

To deal with the decoy state method with statistical fluctuation, we need bring in the expected value of observed variable *S*_*lr*_, *T*_*lr*_, as the form of 〈*S*_*lr*_〉 and 〈*T*_*lr*_〉, so the estimation equations are:

7 $$\begin{array}{c}{s}_{11}\ge {\underline{s}}_{11}( {\mathcal H} )=\frac{[{a}_{1}^{\text{'}}{b}_{2}^{\text{'}}\langle {S}_{xx}\rangle +{a}_{1}{b}_{2}{a}_{0}^{\text{'}}\langle {S}_{oy}\rangle +{a}_{1}{b}_{2}{b}_{0}^{\text{'}}\langle {S}_{yo}\rangle ]-[{a}_{1}{b}_{2}\langle {S}_{yy}\rangle +{a}_{1}{b}_{2}{a}_{0}^{\text{'}}{b}_{0}^{\text{'}}\langle {S}_{oo}\rangle ]-{a}_{1}^{\text{'}}{b}_{2}^{\text{'}} {\mathcal H} }{{a}_{1}{a}_{1}^{\text{'}}({b}_{1}{b}_{2}^{\text{'}}-{b}_{1}^{\text{'}}{b}_{2})},\\ {e}_{11}\le {\bar{e}}_{11}( {\mathcal H} )=\frac{\langle {T}_{xx}\rangle - {\mathcal H} \mathrm{/2}}{{a}_{1}{b}_{1}{ {\mathcal H} }_{11}},\\ {\rm{and}}\, {\mathcal H} ={a}_{0}\langle {S}_{ox}\rangle +{b}_{0}\langle {S}_{xo}\rangle -{a}_{0}{b}_{0}\langle {S}_{oo}\rangle \mathrm{.}\end{array}$$

In these two estimations, we choice *s*_11_ and *e*_11_ as the functions of ${\mathcal H}$, which is the common part of them. (The detail prove of this method can be seen in our previous work^58^). By using this kinds of estimations, the final key rate is also the functions of ${\mathcal H}$. Then through scanning ${\mathcal H}$ in the interval giving by the statistical fluctuation of certain failure probability, the minimum value of $R( {\mathcal H} )$ is final key rate. And it’s much better than the result of treat statistical fluctuation in *s*_11_ and *e*_11_ respectively.

So we have,

8 $$R=\mathop{{\rm{m}}{\rm{i}}{\rm{n}}}\limits_{ {\mathcal H} \,\in \, {\mathcal I} } {\mathcal R} ( {\mathcal H} \mathrm{).}$$

And $$ {\mathcal I} =[h-\delta ,\,h+\delta ]$$ with

9 $$\begin{array}{rcl}h & = & {a}_{0}{S}_{ox}+{b}_{0}{S}_{xo}-{a}_{0}{b}_{0}{S}_{oo}\\ \delta  & = & {a}_{0}\gamma \sqrt{\frac{{S}_{ox}}{{N}_{ox}}}+{b}_{0}\gamma \sqrt{\frac{{S}_{xo}}{{N}_{xo}}}+{a}_{0}{b}_{0}\gamma \sqrt{\frac{{S}_{oo}}{{N}_{oo}}}\end{array}$$

This result is obtained by the theory of statistical fluctuation, *γ* is the parameter decided by the failure probability.(For example, if we choice the failure probability as 1*e* − 7, then *γ* = 5.3)

The other advantage of our method propose in refs^58,72^ is the joint-treatment of the statistical fluctuation, which allow us to get the expect values in the estimations of ${\underline{s}}_{11}$ and ${\bar{e}}_{11}$ by the following constraints:

10 $$\begin{array}{c}{N}_{lr}{S}_{lr}+\gamma \sqrt{{N}_{lr}{S}_{lr}}\ge {N}_{lr}\langle {S}_{lr}\rangle \ge {N}_{lr}{S}_{lr}-\gamma \sqrt{{N}_{lr}{S}_{lr}};\,{\rm{f}}{\rm{o}}{\rm{r}}\,{\rm{a}}{\rm{n}}{\rm{y}}\,lr\in {\mathscr{D}}\\ {N}_{yo}\langle {S}_{yo}\rangle +{N}_{oy}\langle {S}_{oy}\rangle \ge {N}_{yo}{S}_{yo}+{N}_{oy}{S}_{oy}-\gamma \sqrt{{N}_{yo}{S}_{yo}+{N}_{oy}{S}_{oy}}\\ {N}_{xx}\langle {S}_{xx}\rangle +{N}_{yo}{\langle {S}_{yo}\rangle }_{+}{N}_{oy}\langle {S}_{oy}\rangle \ge {N}_{xx}{S}_{xx}+{N}_{yo}{S}_{yo}+{N}_{oy}{S}_{oy}-\gamma \sqrt{{N}_{xx}{S}_{xx}+{N}_{yo}{S}_{yo}+{N}_{oy}{S}_{oy}}\\ {N}_{yy}\langle {S}_{yy}\rangle +{N}_{oo}\langle {S}_{oo}\rangle \le {N}_{yy}{S}_{yy}+{N}_{oo}{S}_{oo}+\gamma \sqrt{{N}_{yy}{S}_{yy}+{N}_{oo}{S}_{oo}}\end{array}$$

and

11 $${N}_{ox}{S}_{ox}+{N}_{xo}{S}_{xo}+\gamma \sqrt{{N}_{ox}{S}_{ox}+{N}_{xo}{S}_{xo}}\ge {N}_{xo}\langle {S}_{xo}\rangle +{N}_{ox}{\langle {S}_{ox}\rangle }_{ {\mathcal L} }\ge {N}_{ox}{S}_{ox}+{N}_{xo}{S}_{xo}-\gamma \sqrt{{N}_{ox}{S}_{ox}+{N}_{xo}{S}_{xo}}$$
